# Involvement of Cyclin K Posttranscriptional Regulation in the Formation of *Artemia* Diapause Cysts

**DOI:** 10.1371/journal.pone.0032129

**Published:** 2012-02-21

**Authors:** Yang Zhao, Xia Ding, Xiang Ye, Zhong-Min Dai, Jin-Shu Yang, Wei-Jun Yang

**Affiliations:** 1 Key Laboratory of Conservation Biology for Endangered Wildlife of the Ministry of Education and College of Life Sciences, Zhejiang University, Hangzhou, Zhejiang, People's Republic of China; 2 College of Life Sciences, Nanchang University, Nanchang, Jiangxi, People's Republic of China; 3 Institute of Developmental and Regenerative Biology, College of Life and Environmental Sciences, Hangzhou Normal University, Hangzhou, Zhejiang, People's Republic of China; University of South Florida College of Medicine, United States of America

## Abstract

**Background:**

*Artemia* eggs tend to develop ovoviviparously to yield nauplius larvae in good rearing conditions; while under adverse situations, they tend to develop oviparously and encysted diapause embryos are formed instead. However, the intrinsic mechanisms regulating this process are not well understood.

**Principal Finding:**

This study has characterized the function of cyclin K, a regulatory subunit of the positive transcription elongation factor b (P-TEFb) in the two different developmental pathways of *Artemia*. In the diapause-destined embryo, Western blots showed that the cyclin K protein was down-regulated as the embryo entered dormancy and reverted to relatively high levels of expression once development resumed, consistent with the fluctuations in phosphorylation of position 2 serines (Ser2) in the C-terminal domain (CTD) of the largest subunit (Rpb1) of RNA polymerase II (RNAP II). Interestingly, the cyclin K transcript levels remained constant during this process. *In vitro* translation data indicated that the template activity of cyclin K mRNA stored in the postdiapause cyst was repressed. In addition, *in vivo* knockdown of cyclin K in developing embryos by RNA interference eliminated phosphorylation of the CTD Ser2 of RNAP II and induced apoptosis by inhibiting the extracellular signal-regulated kinase (ERK) survival signaling pathway.

**Conclusions/Significance:**

Taken together, these findings reveal a role for cyclin K in regulating RNAP II activity during diapause embryo development, which involves the post-transcriptional regulation of cyclin K. In addition, a further role was identified for cyclin K in regulating the control of cell survival during embryogenesis through ERK signaling pathways.

## Introduction

RNA polymerase II (RNAP II) is a key enzyme involved in the synthesis of mRNA, and interruption of its function triggers apoptosis in human cells and induces abnormality in developing embryos [1], [2]. Its activation largely depends on phosphorylation of the C-terminal domain (CTD) of its largest subunit (Rpb1) during transcription [3]. The CTD contains repeats of a seven-amino-acid motif (heptapeptide repeats), which is conserved from yeasts to mammals, although the number of repeats varies [3]–[5]. Serine residues in the consensus motif are phosphorylated by diverse kinases during the processes of transcription [6] and pre-mRNA processing [7]. Positive transcription elongation factor b (P-TEFb) is of great significance in the transcription process, since it facilitates the transition from abortive to productive elongation by phosphorylating position 2 serines (Ser2) on the heptapeptide repeats [4], [5], [8]–[11].

P-TEFb, which comprises a kinase subunit, CDK9, and its cyclin partner, cyclin T, has attracted much attention because of roles in diverse biological processes such as embryonic development, cell differentiation and HIV-1 replication in humans [2], [12], [13]. Cyclin K, the latest member discovered to be associated with CDK9, is less well studied. Although one report showed that a CDK9-cyclin K complex participates directly in the DNA damage response [14], its role as a component of P-TEFb *in vivo* is uncertain. An *in vitro* kinase assay proved that the CDK9-cyclin K complex could functionally substitute the CDK9-cyclin T complex to phosphorylate CTD on Rpb1 of RNAP II without regard to the lower activity [15], [16]; however, other research suggested that cyclin K is not involved in DNA transcription *in vivo* [17], [18].

The brine shrimp, genus *Artemia*, evolved the extraordinary ability to reproduce via encysted gastrula embryos (cysts), which are able to cope with harsh environments including anoxia, high salinity, high pH, and major changes in the ionic composition and temperature of the surrounding environment. The cyst, which has a barely detectable metabolic rate and exists in a state of obligate dormancy called diapause, can survive for extremely long periods [19]. This diapause status can be terminated by exposure to specific environmental stimuli and the embryo then undergoes postdiapause development, eventually emerging as a fully formed nauplius larva in a suitable environment. This development process is very complicated and involves a large number of internal events, all of which are coupled to the expression of a large number of different genes.

To understand the mechanism of diapause completely, it is necessary to investigate the action of RNAP II, since transcription is inhibited in diapause embryos [19]. RNAP II has been purified at different stages of *Artemia* development [20]–[22], and although a detectable level of RNAP II is present in the diapause embryo, its activity is less than 10% of that in nauplii. Most of it is present in a free form which is not bound to chromatin and becomes actively engaged in transcription upon development [19]. Multiple factors are involved in this transition phase, and the enzyme has a complex composition and is regulated by multiple mechanisms. Its largest subunit, Rpb1, with a molecular mass of Mr 205,000 in developing cysts, is converted into a polypeptide of Mr 172,000 in larvae. This proteolytic modification is thought to be the mechanism involved in regulating RNAP II activity upon larval development [23]. A more direct regulator of RNAP II activity, known as the S protein, has been isolated from the cytosol of dormant and developing cysts. It is known to activate RNAP II through its action on the enzyme rather than on the DNA template and decreases in the period of pre-emergence and early larval development. However, the mechanism of activation is unknown [24].

In the present study, we identified a cyclin K homolog from an *Artemia parthenogenetica* cDNA library and explored its functions in the two different developmental pathways of *Artemia*. Transcription levels, tested by semiquantitative reverse transcription-PCR, showed that cyclin K is most abundant in the postdiapause developmental embryos, suggesting that it plays a specific role during embryonic development. In addition, cyclin K was studied further in the oviparous developmental pathway that includes a long period of diapause. Repression of cyclin K in diapause embryos was related to a specific mechanism that reduces the template activity of mRNA and, hence, inhibits the phosphorylation of RNAP II. Western blot analysis and immunofluorescence staining of nuclei indicated that increasing levels of cyclin K play an essential role in postdiapause development by regulating the phosphorylation of CTD Ser2 of RNAP II. Additionally, RNA interference (RNAi) *in vivo* knockdown of cyclin K in early embryos provided direct evidence that phosphorylation of CTD Ser2 was cyclin K-dependent and showed that a lack of cyclin K induced apoptosis by inhibiting ERK-mediated survival signaling.

## Materials and Methods

### Animal Culture and Sample Collection

*A. parthenogenetica* from Gahai Lake, China, were gifted by Prof. Feng-Qi Liu (Nankai University, Tianjin, China). Specimens are separated into two groups and cultured in different conditions. One group was cultured in 8% artificial seawater (Blue Starfish, Hangzhou, China) with a 5-h light cycle per day. Under these conditions, the majority reproduced oviparously and released encysted diapause embryos. The other group was reared in 4% artificial seawater with a 16-h light cycle per day and almost all specimens reproduced ovoviviparously and yielded swimming nauplii. Both groups were reared at 28°C and fed with *Chlorella* powder (Fuqing King Dnarmsa Spirulina Co. Ltd., Fuqing, China) every 2 days.

*Artemia* with oviparously or ovoviviparously developing embryos were classified by observing the shell gland morphology as described by Liang and MacRae [25]. Samples of the reproductive tract were collected at each developmental stage, and ovisac isolation performed according to the method of Liu et al [26].

Encysted embryos were collected and stored dry at 25°C as examples of diapause embryos, and were used within 2 weeks. The hatchability was below 10% in artificial sea-water (3%) under continuous light at 25°C. Other gathered diapause embryos were dehydrated in saturated sodium chloride solution for 24 h and then stored at −20°C to prepare a sample of postdiapause embryos. Postdiapause cysts were hydrated at 4°C for 5 h and then incubated in artificial sea-water (3%) under continuous light at 25°C. Samples were taken at 0, 2, 4, 8, 10, 12 and 14 h, and at the free-swimming nauplius stage. All samples were snap-frozen in liquid nitrogen and stored at −80°C until required for RNA and protein preparation.

### Semiquantitative Reverse Transcription-PCR

Total RNA was extracted from each sample using TRIzol reagent (Invitrogen, Carlsbad, CA, USA) according to the manufacturer's instructions. First-strand cDNAs were synthesized from 1 µg of total RNA using M-MLV Reverse Transcriptase (Promega, Madison, WI, USA) in a 12.5-µl reaction. Cyclin K (GenBank™ accession number JQ085432), Rpb1 (GenBank™ accession number U10331) and α-tubulin cDNA fragments (GenBank™ accession number AF427598) were amplified separately in 25-µl reactions using 1 µl of each reverse transcription product as a template. The primers used are shown in Table 1 (cycKQF and cycKQR for cyclin K; Rpb1F and Rpb1R for Rpb1; TubF and TubR for α-tubulin).

**Table 1:** Nucleotide sequences and positions of primers used in polymerase chain reactions.

Primer	Length (bp)	Position	Direction	Sequence (5′-3′)
cycKQF	20	41–60	F	TACGAAAGACCCCATCAGCA
cycKQR	20	235–254	R	AGAAAGAGGCAGCAACAAGC
cycKF	22	10–31	F	ATGCCTTGCTGGTATTATGATA
cycKR	22	1068–1089	R	ATATGGCGGTCTTGGTGGTTAA
cycKiF	28	550–569	F	GCTCTAGA TGGGAGCCGGAAATAATAGC
cycKiR	29	1032–1051	R	CCGGAATTC TGGGGTAAAAAGAAGCAGTG
TubF	20	532–551	F	TCTACTGCCGTTGTTGAGCC
TubR	20	694–713	R	ATGGAGGAAACGATTTGACC
Rpb1F	20	821–840	F	CAGCGCGTACTGTCATTACC
Rpb1R	20	1135–1154	R	CTGTGGCCCATCATAGACAT

F and R indicate the forward and reverse directions, respectively. The underlined regions represent the adscititious recognition sequences of restriction endonucleases.

### Western Blotting

Proteins were extracted from each sample using TRIzol reagent (Invitrogen, Carlsbad, CA, USA) according to the manufacturer's instructions and quantified using the Bradford method [27]. From each sample, 25 µg of protein were separated by SDS-PAGE and transferred to polyvinylidene difluoride membranes (Millipore, Bedford, MA, USA). Membranes were incubated with a primary antibody overnight at 4°C and detection was performed using the BM Chemiluminescence Western Blotting Kit (Roche, Mannheim, Germany). A peptide, located at the C-terminus of cyclin K (amino acids [aa] 346 to 359), was chemically synthesized and used to immunize rabbits to obtain the antibody (Hangzhou HuaAn Biotechnology Company, Hangzhou, China). Anti-RNA polymerase II CTD repeat YSPTSPS (phospho S2) [H5] antibody (ab24758) and anti-phospho-RSK antibody (ab10695) were purchased from Abcam (Cambridge, UK); anti-phospho-ERK1/2 antibody (9101) and anti-ERK1/2 antibody (9102) were purchased from Cell Signaling Technology (Danvers, MA, USA); anti-phospho-histone H3 antibody (1173-1) and anti-α-tubulin (1878-1) antibodies were purchased from Epitomics, Inc (Burlingame, CA, USA).

### Colocalization of Cyclin K with Phosphorylated RNAP II

Embryos incubated for 14 h were homogenized using Dounce homogenizers in buffer K (10 mM Hepes pH 7.2, l mM EDTA, 50 mM NaCl, pH 6.0 or 8.0) and cell fractionation performed as previously described [28]. Cyclin K and phosphorylated CTD Ser2 of RNAP II were detected in both supernatants and pellets as described above. H3 and α-tubulin (Epitomics, Inc, Burlingame, CA, USA) were detected simultaneously to determine the purity of the fractions.

Nuclei from the 14-h incubated embryos were prepared as described [29] and fixed in 4% (w/v) paraformaldehyde. Immunofluorescence staining was performed as described [30]. Cyclin K (1∶100) and H5 (1∶100) were used as the primary antibodies. The secondary antibodies were FITC-conjugated goat anti-mouse IgM (1∶200; Santa Cruz Biotechnology, Inc, Santa Cruz, CA, USA) and TRITC-conjugated goat anti-rabbit IgG (1∶200; Hangzhou HuaAn Biotechnology Company, Hangzhou, China). After incubation with the secondary antibody and a rinse in PBS, the nuclei were counterstained with 4′,6-diamidino-2-phenylindole (DAPI) (Beyotime, Shanghai, China). Slides were examined with a Nikon ECLIPSE TE200-S microscope (Nikon, Tokyo, Japan).

14-h incubated embryos were decapsulated using antiformin [28], and homogenized using Dounce homogenizers in precooled FA lysis buffer ((50 mM Hepes pH 7.5, 140 mM NaCl, 1 mM EDTA, 1% Triton X-100, 0.1% sodium deoxycholate and protease inhibitors) on ice. Cell lysates were clarified by centrifugation and precleared by incubation for 1–2 h with Protein A Sepharose beads (Invitrogen, Carlsbad, CA, USA). The precleared supernatants were then incubated overnight at 4°C with anti-cyclin K antibody or anti-SGEG2a (which has been identified as one component of the cysts shell) antibody [31] as a negative control. The proteins were immunoprecipitated using protein A Sepharose beads (Invitrogen, Carlsbad, CA, USA) and then analysed by western blot.

### Oligo(dT)-Cellulose Affinity Chromatography and *In Vitro* Translation

Postdiapause cysts (10 g dry weight) and 8-h incubated embryos were decapsulated using antiformin [28] and ground to a fine powder in liquid nitrogen after being thoroughly washed in distilled water. The powder was transferred to precooled buffer J (10 mM Hepes pH 7.2, 5 mM MgCl_2_, 50 mM NaCl) [32] containing 0.5 mg/ml heparin and 150 mM sucrose. The post-mitochondrial supernatant was prepared by centrifugation as previously described [32]–[34]. These supernatants were adjusted to 250 mM NaCl and then separated on an oligo(dT)-cellulose chromatography column (Sigma-Aldrich, St. Louis, MO, USA). After extensive washing with buffer L (10 mM Hepes pH 7.2, 250 mM NaCl) [31], the cellulose was eluted with 10 mM Hepes (pH 7.2) at room temperature and concentrated by ultrafiltration [32]. The eluate was used as a template for protein synthesis and for RNA extraction to do northern blot. The *in vitro* translation assay was performed using the RTS 100 Wheat Germ CECF Kit (5PRIME, Gaithersburg, MD, USA).

### Northern Blotting

For postdiapause and 8-h incubated cysts, 8 µg mRNA were separated by 1.0% agarose gel electrophoresis and then transferred to a positively-charged nylon membrane (Millipore, Bedford, MA, USA). After pre-hybridization at 42°C for 1 h, the membrane was hybridized at 42°C overnight with a DIG-labeled probe to detect cyclin K (amplified using the primers cycKF and cycKR [Table 1]) or a DIG-labeled probe to detect tubulin (amplified using the primers TubF and TubR [Table 1]). After extensive washing, hybridized probes were visualized using a DIG chemiluminescent detection system (Roche, Mannheim, Germany).

### RNA Interference

A fragment of cyclin K, amplified using the primers, cycKiF and cycKiR (Table 1), was subcloned into the plasmid pET-T7 [35], [36] between the *Xba*I and *Eco*RI sites. Plasmids expressing *green fluorescent protein* (GFP) dsRNA as a negative control were constructed as described [26]. The recombinant plasmids were transformed into *Escherichia coli* HT115 and the dsRNAs were produced and purified as described [36]. Cyclin K and GFP dsRNAs (500 ng) were injected separately into the reproductive segments of *Artemia* at stage instar XII (before ovarian development) using the UltraMicroPump II (World Precision Instruments Inc, Sarasota, FL, USA) equipped with a Micro4™ microsyringe pump controller (World Precision Instruments Inc, Sarasota, FL, USA). The injected individuals were cultured under the conditions already described for rearing ovoviviparous *Artemia*.

### TUNEL Assay

Adult *Artemia* with embryos of each stage were anesthetized on ice, snap-freezing in liquid nitrogen, and embedded in Tissue Tek (Sakura Finetechnial Co. Ltd, Tokyo, Japan). Frozen sections, each 8-µm thick, were prepared using a frozen ultra-microtome. The terminal deoxynucleotidyl transferase dUTP nick end-labeling (TUNEL) assay was performed using the DeadEnd Colorimetric TUNEL System (Promega, Madison, WI, USA) according to the manufacturer's instructions.

## Results

### Characterization and mRNA Expression Pattern of Cyclin K and Rpb1 in the two Developmental Pathways of *Artemia*

**Figure 1:**
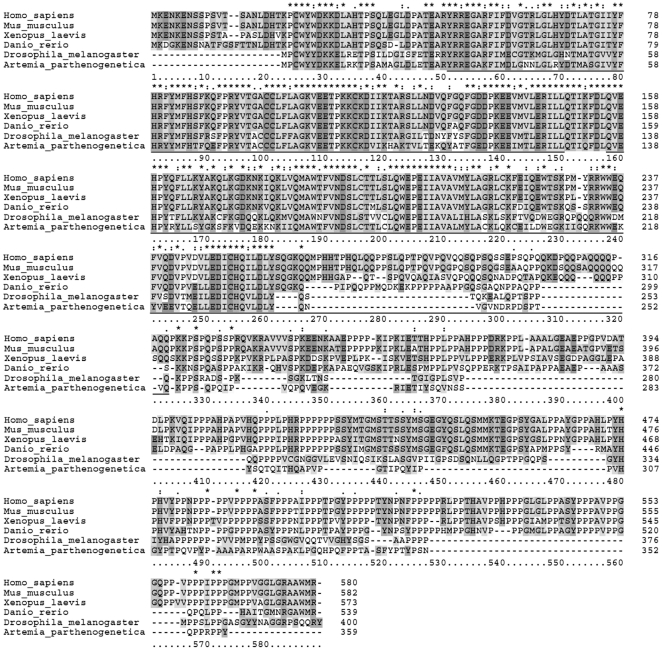
Sequence comparison with other species and phylogenetic analysis of *Artemia* cyclin K. Alignment of *Artemia* cyclin K with human (NP_001092872.1), mouse (NP_033962.2), *Xenopus* (NP_001089373.1), zebrafish (NP_001157251.1) and *Drosophila* (NP_788082.1). Numbers on the right side refer to the amino-acid position. Asterisks indicate conserved residues in all sequences; a single dot indicates semi-conserved substitutions and a double dot indicates conserved substitutions. The sequences underlined refer to the two cyclin boxes in *Artemia* cyclin K.

An *A. parthenogenetica* cDNA library was constructed from both oviparous and ovoviviparous whole animals [37] and sequencing performed. One transcript containing a full-length open reading frame encoding a 359-aa protein was identified. Sequence analysis showed it contained two typical cyclin boxes (aa 29 to 128 and aa 136 to 254, Fig. 1) and the deduced amino acid sequence comparison results showed that *Artemia* cyclin K had 52.9%, 54.0%, 53.6%, 52.1% and 52.4% sequence identity with cyclin K of human, mouse, *Xenopus*, zebrafish, and *Drosophila* respectively (Fig. 1), confirming that the transcript identified was an *Artemia* cyclin K ortholog. The nucleotide sequence of this cyclin K encoding cDNA was submitted to GenBank™ under the accession number JQ085432.

**Figure 2:**
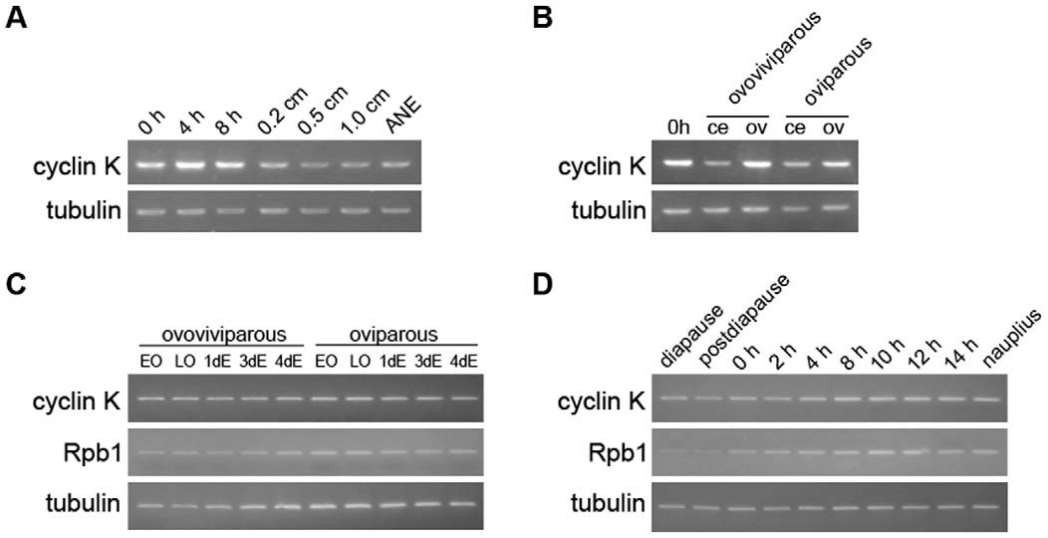
Semiquantitative RT-PCR analysis of cyclin K expression in *Artemia*. (**A**) Cyclin K expression during *Artemia* development: postdiapause development stages (0, 4 and 8 h) represent postdiapause cysts incubated for 0, 4 and 8 h, respectively. Larval and post-larval development stages (0.2, 0.5 and 1.0 cm) represent larva of body length 0.2, 0.5 and 1.0 cm, respectively. ANE: adults without eggs. (**B**) Cyclin K expression in cephalothorax (ce) and ovisacs (ov) of both oviparous and ovoviviparous animals. 0 h is as the same sample as 0 h in (A) and represents postdiapause cysts incubated for 0 h. (**C**) Expression of cyclin K and Rpb1 in oocytes and embryos of both oviparous and ovoviviparous developmental pathways. EO, early oocytes; LO, late oocytes; 1dE, 3dE and 4dE represent embryos having entered the uterus for 1 day, 3 days and 4 days, respectively. (**D**) Expression of cyclin K and Rpb1 during the hatching process of diapause embryos (which includes the diapause embryo, postdiapause embryo, 0- to 14-h incubated embryos and nauplius); α-tubulin was used as a loading control.

Cyclin K mRNA was detected in different tissues and developmental stages of *Artemia*. Semiquantitative RT-PCR showed that the cyclin K transcript was detectable throughout the life-cycle of *Artemia*, and was most abundant during embryonic development (Fig. 2A). In addition, it showed a higher level of expression in the ovisac (Fig. 2B), consistent with the fact that cyclin K is abundantly expressed in developing germ cells of mouse [15]. However, no significant differences in expression were detected between embryos in the two developmental pathways or during the hatching process of the encysted embryo (Fig. 2C and 2D).

Primers were designed according to the sequence of *A. salina* Rpb1 and semiquantitative RT-PCR was also performed to examine the Rpb1 gene expression level in the embryos of the two developmental pathways and during the hatching process of the encysted embryo. The results showed that there were no significant differences in Rpb1 expression between the two developmental pathways; however, Rpb1 was expressed at relatively low levels in diapause and postdiapause embryos, and its expression was restored as development was initiated (Fig. 2C and 2D).

### Expression and Localization of Cyclin K Protein and Activated RNAP II in the Development of Diapause-Destined Embryos

**Figure 3:**
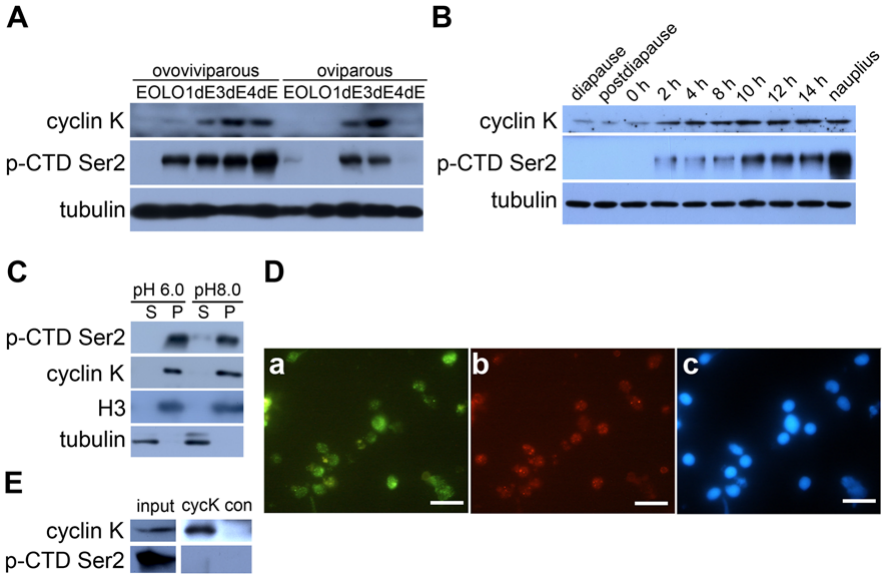
Cyclin K protein expression and phosphorylation of RNAP II in different developmental phases and their subcellular localization in *Artemia* embryos. (**A**) Cyclin K protein expression and CTD Ser2 phosphorylation of RNAP II in the two developmental pathways. EO, early oocytes; LO, late oocytes; 1dE, 3dE and 4dE represent embryos entering uterus for 1 day, 3 days and 4 days, respectively. (**B**) Cyclin K protein expression and CTD Ser2 phosphorylation of RNAP II during the hatching process of diapause embryos (includes diapause embryo, postdiapause embryo, 0- to 14-h incubated embryos and nauplius); α-tubulin was used as a loading control. (**C**) Supernatant (S) and pellet (P) fractions were prepared using buffer K (pH 6.0 or 8.0) from 14-h incubated embryos. Cyclin K and CTD Ser2 phosphorylation of RNAP II were detected by Western blotting. Tubulin and H3 were also examined to indicate the purity of the different extracts. (**D**) Immunofluorescence staining of nuclei from 14-h incubated embryos confirmed that cyclin K co-localizes with phosphorylated RNAP II in nuclei. a, cyclin K; b, phosphorylation of CTD Ser2; c, DAPI stain. The bars represent 10 µm. (**E**) Cyclin K and its associated factors (anti-cyclin K immunoprecipitates) were affinity purified from the 14-h incubated embryos and analysed by western blot. Another polyclonal antibody produced in rabbit (anti-SGEG2a) was used in a parallel procedure for control (con). The input loading quantity was 1/100 of the total supernatants.

Before postdiapause development resumes, RNA synthesis ceases totally in encysted embryos [19]. Considering that cyclin K can activate transcription through RNAP II *in vitro*, cyclin K and the phosphorylation of CTD Ser2 of RNAP II were investigated simultaneously in the two developmental pathways and in the hatching process of encysted embryos. A rabbit polyclonal antibody was generated against a synthetic peptide based on the partial sequence of *A. parthenogenetica* cyclin K, and Western blotting revealed that the cyclin K protein was hardly detected in oocytes and then, it accumulated along the embryo developing in both two developmental pathways. Moreover, it was specifically down-regulated in the oviparous embryo one day before release to the environment and reverted to a relatively high level when development resumed (Fig. 3A and 3B). The RNAP II phosphorylation during the development of diapause-destined embryos was examined using an H5 mouse monoclonal antibody. It has been used to detect CTD Ser2 phosphorylation in many species ranging from yeast to humans [4], although a previous study reports that H5 also shows some cross-reactivity with CTD phosphorylation at Ser5 [38]. As speculated, Western blot analysis showed that CTD Ser2 phosphorylation appeared when cyclin K was expressed and that the phosphorylation level altered along with cyclin K expression levels in both developmental pathways, except that it had high phosphorylation level in the ovoviviparous late oocytes whereas cyclin K had little expression in this sample (Fig. 3A and 3B).

Next, we extracted pellet and supernatant proteins of 14-h-incubated cysts according to the method previously established [28]. The pellet fraction contains nuclei and yolk platelets, while the supernatant contains cytoplasmic proteins. As shown in Fig. 3C, cyclin K and CTD Ser2 phosphorylation of RNAP II were mainly detected in the pellet extracts, with little or no expression or phosphorylation in the supernatant extracts. Immunofluorescence staining of nuclei further confirmed that cyclin K and CTD Ser2 phosphorylated RNAP II were co-localized in the nucleus. However, the cyclin K staining degree were not proportional to the staining of phospho-RNAP II (Fig. 3D). Moreover, the phospho-RNAP II could not be detected in the immunoprecipitates by cyclin K antibody (Fig. 3E). All of these results suggested that cyclin K is a possible key factor involved in regulating the activity of RNAP II during diapause-destined embryo development, even if cyclin K could not bound to CTD Ser2 phosphorylated RNAP II.

### Cyclin K is regulated at the Post-transcriptional Level in *Artemia* Diapause Embryos

**Figure 4:**
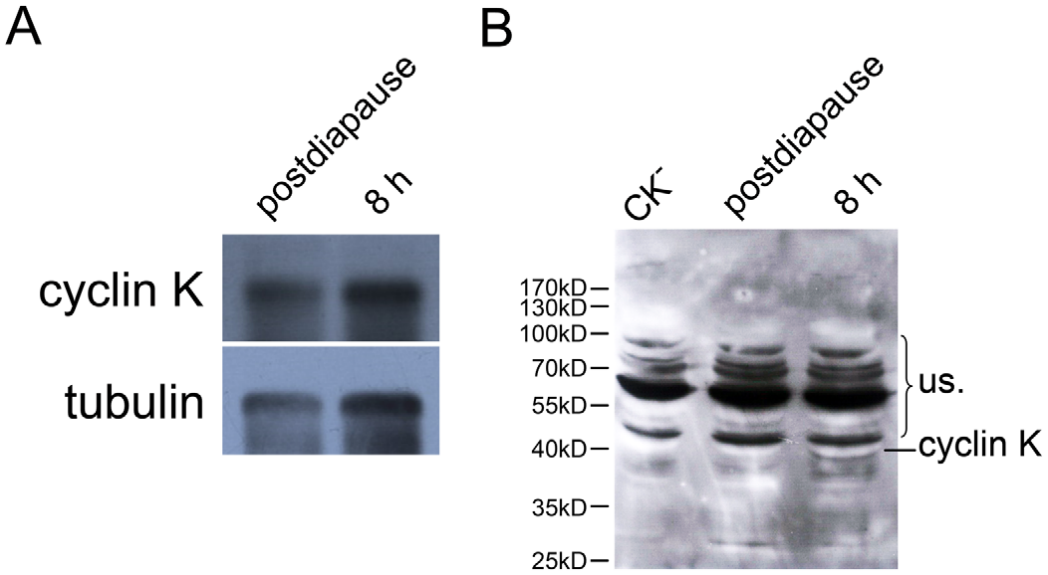
The template activity of cyclin K mRNA is repressed in encysted embryos. Poly(A)-containing mRNA was purified from postdiapause and 8-h incubated (8 h) embryos using oligo(dT)-Cellulose, and *in vitro* translation was performed using each purified mRNA as a template. (**A**) Cyclin K was detected in each purified mRNA sample by Northern blotting. (**B**) Detection of cyclin K *in vitro* translation product by Western blotting. NS: nonspecific bands. CK^−^, DEPC-treated water was used as template for the *in vitro* translation control. The molecular weight was shown on the left.

Previous research reports that the amount of poly(A)-containing mRNAs are associated with one translational inhibitor RNA or protein p38 in dormant embryos and present in a repressed form [32], [39]. Since we observed that the cyclin K protein declined in encysted embryos (Fig. 3A and 3B) whereas mRNA remained at a constant level (Fig. 2C and 2D), the template activity of cyclin K mRNA was tested. Total poly(A)-containing mRNA was purified from postdiapause and 8-h incubated embryos using oligo(dT)-cellulose columns and *in vitro* translation was performed. Northern blotting detected cyclin K in both postdiapause and 8-h incubated embryos purified mRNA by oligo(dT)-cellulose chromatography (Fig. 4A). However, the cyclin K protein was not detected following *in vitro* translation of the purified mRNA from diapause embryos, but was detectable in 8-h incubated embryos (Fig. 4B). This indicates that cyclin K was repressed in postdiapause cysts and is somehow reactivated after the initiation of development.

### Lack of Cyclin K Inhibits RNAP II Activity and Induces Apoptosis Mediated by ERK Pathways in Early Embryonic Development of *Artemia*

**Figure 5:**
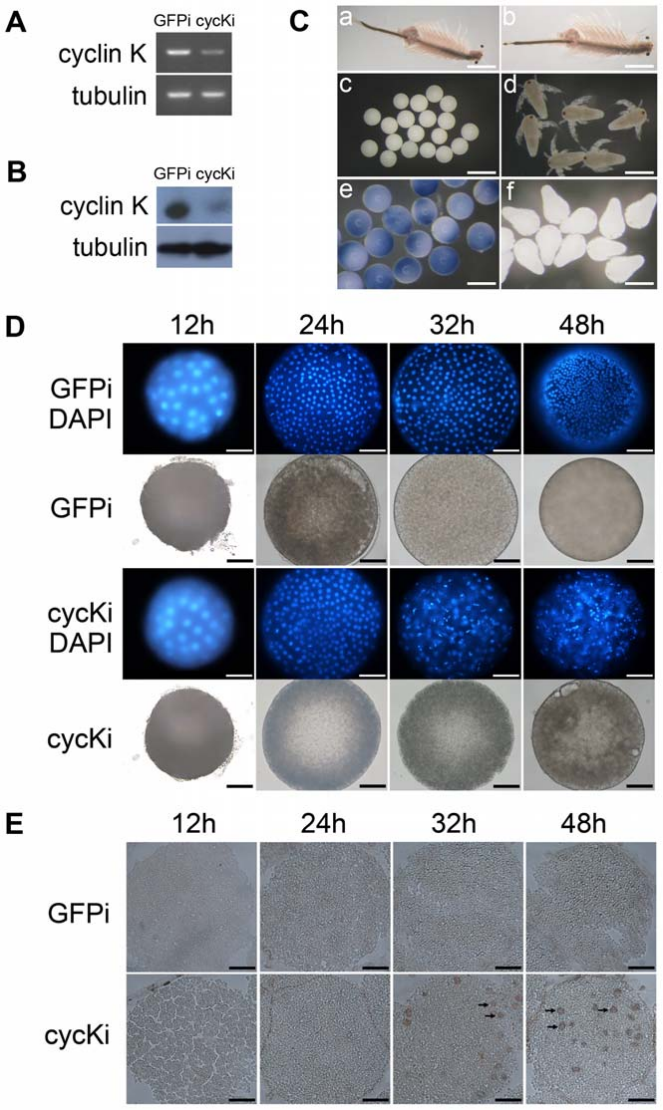
Knockdown of cyclin K in developing embryos. (**A**) Semiquantitative RT-PCR analysis of cyclin K mRNA levels in control (GFPi) and test (cycKi) groups. (**B**) Western Blot analysis of cyclin K protein levels in control (GFPi) and test (cycKi) groups. (**C**) Morphology of adult *Artemia*: cyclin K RNAi (a) and GFP RNAi (b); Offspring produced by cyclin K RNAi (c) and GFP RNAi (d) *Artemia*; Trypan Blue staining of embryos entering utrus for four days reproduced by cyclin K RNAi (e) and GFP RNAi (f) *Artemia*. The bars in (a) and (b) represent 30 mm. The bars in (c) represent 215 µm. The bars in (d–f) represent 150 µm. (**D**) DAPI staining of early development embryos. The samples are eggs having entered the uterus for 12, 24, 32 and 48 h respectively. The upper two panels are GFP RNAi groups (GFPi); the lower two panels are cyclin K RNAi groups (RNAi). (**E**) TUNEL assay of embryos at the same stages of development as shown in D, 8-µm frozen sections were prepared and DNA strand breaks were detected by the TUNEL assay. Arrows indicated positive signals in cyclin K RNAi embryos. The bars in both (D) and (E) indicate 35 µm.

P-TEFb (CDK9/cyclin T) is required for the transcription of early embryonic genes [2], [12], [13]. Considering that cyclin K can form a complex with CDK9 to function as P-TEFb *in vitro*, whether it is involved in early embryo development was investigated. Double-stranded RNA (dsRNA), based on the cyclin K sequence, was injected into *Artemia* at instar XII stage as previously described [40], and GFP dsRNA was injected as a control. Both mRNA and protein levels of cyclin K were substantially depleted (Fig. 5A and 5B), suggesting a severe reduction in cyclin K expression *in vivo*. The morphology of adult and embryonic *Artemia* were examined, and although the *cyclin K* RNAi maternal *Artemia* showed no significant abnormal phenotypes compared with the control group (Fig. 5C, a and b), they released embryos that could not develop into nauplii (Fig. 5C, c and d), and Trypan Blue staining of the embryos entering uterus for four days confirmed that cells in RNAi embryos had lost viability (Fig. 5C, e and f). DAPI staining showed that the *cyclin K* RNAi embryo was normal for the first 24 h after it entered the uterus, whereas the nuclei were disorganized at the blastula stage (32 h after embryos entered the uterus) (Fig. 5D). A TUNEL assay confirmed that the cells in the RNAi embryos were apoptotic (Fig. 5E).

**Figure 6:**
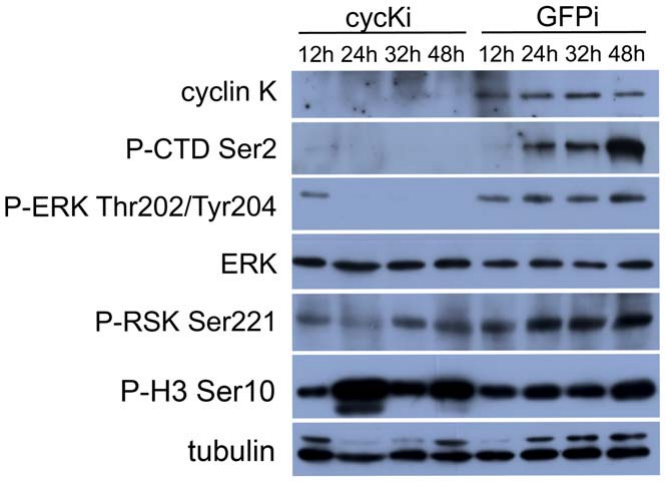
Depletion of cyclin K eliminated the phosphorylation of RNAP II and ERK. Ovoviviparous *Artemia* were divided into two groups with injection of cyclin K dsRNA (cycKi) or GFP dsRNA (GFPi). Embryos from each group were sampled at the same time points as shown in Figure 5D and 5E. The expression level of cyclin K and ERK, RNAP II phosphorylated at CTD Ser2, ERK phosphorylated at Thr202 and Tyr204, RSK phosphorylated at Ser 221 and H3 phosphorylated at Ser10 were detected by Western blotting.

To study the mechanism of apoptosis triggered by the loss of cyclin K further, the signaling pathway was investigated. As speculated, phosphorylation of the RNAP II CTD Ser2 diminished after cyclin K knockdown, indicating an indispensable role for cyclin K in regulating RNAP II activity. The phosphorylation of ERK was also clearly down-regulated in cyclin K RNAi embryos, whereas the phosphorylation of RSK, its downstream kinase, was not affected. The phospho-H3 level was not reduced, indicating that cell division was not influenced by the depletion of cyclin K (Fig. 6). These results indicated that knockdown of cyclin K induced apoptosis by repressing the ERK pathway, although the downstream effector was not RSK.

## Discussion

Cyclin K was first identified by its ability to rescue a lethal deletion of the G1 cyclin genes in *S. cerevisiae* [15]. Subsequent studies focused on its CTD kinase activity with CDK9 *in vitro* [16], [17], while a recent study reported a direct role for the cyclin K-CDK9 complex in maintaining genome integrity in response to replication stress [14]. The results of the present study show that post-transcriptional regulation of cyclin K is involved in the regulation of RNAP II in *Artemia* diapause embryo development, and that cyclin K is involved in cell survival controlled by ERK signaling pathways during early development of *Artemia* embryos.

### Phosphorylation of RNAP II CTD Ser2 *In Vivo* is Cyclin K-Dependent

Previous studies showed that the cyclin K-CDK9 complex phosphorylates the CTD of RNAP II *in vitro*. In the current study, the *in vivo* cyclin K knockdown provided direct evidence that phosphorylation of Ser2 in the RNAP II CTD repeats is cyclin K-dependent.

A study on the structure-function relationship of CDK9 cyclin partners reported that cyclin K lacks the histidine-rich stretch present in the C-terminal domain of cyclin T1, which is regarded as the unphosphorylated CTD binding site [41]. Another report revealed that cyclin K could only activate transcription via RNAP II when tethered to RNA but not to DNA [17]. By contrast, a co-immunoprecipitation assay performed by another group showed that cyclin K is associated with a hypophosphorylated form of RNAP II (IIa) [15]. In our study, Western blot analysis of different portions of the cyst extracts confirmed that cyclin K and the hyperphosphorylated form of RNAP II (IIo), detected by the H5 antibody, were both located in the nuclei of 14-h incubated embryos (Fig. 3C), but immunofluorescence staining of nuclei showed that the cyclin K staining degree were not proportional to the staining of IIo (Fig. 3D), and IIo was not detected in cyclin K immunoprecipitates (IPs) from 14-h incubated embryo extracts (Fig. 3E). From all of these we thus propose a possible association pattern of cyclin K and RNAP II that P-TEFb, which contains cyclin K, is recruited to RNAP IIa with the help of other transcription factors, in particular those that possess RNA binding activity, and then disassociates from IIo to activate a new cycle of transcription.

### A Potential Mechanism for Regulating RNAP II Activity during Postdiapause Development of *Artemia*

RNAP II was purified from *A. salina* and its activity broadly examined at different development stages with respect to its essential role in gene expression [20]–[22], . However, its regulating mechanism is not well understood. In this study, we propose cyclin K as a potential candidate for the regulation of RNAP II activity in the diapause-destined embryo. Consistent with the CTD Ser2 phosphorylation of RNAP II, the cyclin K protein disappeared 1 day before the cysts were released to the environment, then reappeared and increased as development resumed (Fig. 3A and 3B). The *in vivo* knockdown of cyclin K in *Artemia* eliminated the CTD Ser2 phosphorylation of RNAP II and, from the results of these experiments, we assumed that down-regulation of RNAP II activity in dormant cysts may be largely dependent on the reduction in cyclin K expression. Moreover, the maintenance of cyclin K mRNA without translation to the protein in the encysted embryo (Fig. 4B) suggests that, immediately after translation is reactivated, the cyclin K protein is rapidly synthesized so that it can activate CDK9 to phosphorylate RNAP II, which then switches on transcription in the developing embryo. In addition, our findings also show that Rpb1 mRNA levels were reduced in both diapause and postdiapause cysts, suggesting that the regulation of RNAP II occurs at multiple levels.

One interesting finding was that RNAP II has a relatively high level of phosphorylation in late oocytes produced by the ovoviviparous pathway, whereas phosphorylation was hardly detected in diapause-destined oocytes (Fig. 3A). One possible explanation for this is that genes are expressed differentially in oocytes from the two developmental pathways. This hypothesis corresponds to a previous study, which indicates that many genes show differential expression between the two developmental pathways at the oocyte stage [43], [44]. However, RNAP II activity at this stage is not cyclin K-dependent.

### *In Vivo* Knockdown of Cyclin K in *Artemia* Early Embryos Induces Apoptosis at the Blastula Stage

Metazoan early embryonic development is controlled initially by maternal mRNAs and the onset of embryonic transcription occurs at a later developmental stage, ranging from the two-cell stage in mice to cell-cycle 12 to 14 in flies [44]. In the present study, CTD Ser2 phosphorylation of RNAP II was monitored during early embryonic development and the results showed that transcription activity of RNAP II appears around 24 h after the embryo enters the uterus (early blastula stage, GFP control group in Fig. 6).

Previous studies suggest that impairment of RNAP II activity affects normal embryonic development. Loss of cyclin H function, a subunit of the general transcription factor complex, TFIIH, delays the onset of transcription in early zebrafish embryos and induces apoptosis 5 h post-fertilization [45]. In addition, depletion of CDK9 or cyclin T seems to have more severe effects on embryos during early development leading to loss of viability during metamorphosis in flies and at the 100-cell stage in *C. elegans* [2]. In this study, the *in vivo* knockdown of cyclin K in embryos eliminated the CTD Ser2 phosphorylation of RNAP II, which prevented the embryos from developing into nauplius larvae (Fig. 5C). This suggests that cyclin K participates in the activation of embryonic gene transcription by RNAP II and plays an essential role in early embryonic development. Adult *Artemia* with cyclin K knockdown showed no abnormal phenotypes (Fig. 5C, a and b). One explanation for this is that cyclin K is not essential for the activation of RNAP II transcription in the adult, which is supported by the fact that CDK9 can activate RNAP II transcription in combination with other cyclin partners [46].

Previous studies report that inhibition of ERK survival signaling leads to apoptosis in many cell types [47]–[50]. In our knockdown experiments, the ERK phosphorylation was reduced markedly after depletion of cyclin K and, subsequently, apoptosis was induced in developing embryos (Fig. 5E and 6). These data suggest that apoptosis may possibly have been induced through the ERK pathway. This signaling pathway is not RSK-dependent, as RSK was not affected after cyclin K knockdown (Fig. 6). Such a signaling pathway is also hinted at in a recent study, which reported that *in vivo* knockdown of RhoA induced apoptosis in zebrafish embryos through a reduction in the activation of growth-promoting ERK and decreased expression of anti-apoptotic bcl-2 [51].

### Conclusions

In conclusion, the findings reported in this study illustrate the post-transcriptional regulation of cyclin K and its potential role in regulating RNAP II activity in *Artemia* diapause embryo development. Furthermore, we have identified a further role for cyclin K in regulating the control of cell survival through the ERK pathway during *Artemia* development.
